# Default Network Activity Is Associated with Better Performance in a Vigilance Task

**DOI:** 10.3389/fnhum.2017.00623

**Published:** 2017-12-22

**Authors:** Carsten Bogler, Alexander Vowinkel, Paul Zhutovsky, John-Dylan Haynes

**Affiliations:** ^1^Charité – Universitätsmedizin Berlin, corporate member of Freie Universität Berlin, Humboldt-Universität zu Berlin, and Berlin Institute of Health (BIH), Bernstein Center for Computational Neuroscience, Berlin Center for Advanced Neuroimaging, Department of Neurology, and Excellence Cluster NeuroCure, Berlin, Germany; ^2^Berlin School of Mind and Brain and Institute of Psychology, Humboldt-Universität zu Berlin, Berlin, Germany; ^3^SFB 940 Volition and Cognitive Control, Technische Universität Dresden, Dresden, Germany

**Keywords:** vigilance, default network, reaction time, fMRI, sustained attention

## Abstract

When attention has to be maintained over prolonged periods performance slowly fluctuates and errors can occur. It has been shown that lapses of attention are correlated with BOLD signals in frontal and parietal cortex. This raises the question how attentional fluctuations are linked to the fronto-parietal default network. Because the attentional state fluctuates slowly we expect that potential links between attentional fluctuations and brain activity should be observable on longer time scales and importantly also before the execution of the task. In the present study we used fMRI to identify brain activity that is correlated with vigilance, defined as fluctuations of reaction times (RT) during a sustained attention task. We found that brain activity in visual cortex, parietal lobe (PL), inferior and superior frontal gyrus, and supplementary motor area (SMA) was higher when the subject had a relatively long RT. In contrast to our expectations, activity in the default network (DN) was higher when subjects had a relatively short RT, that means when the performance was improved. This modulation in the DN was present already several seconds before the task execution, thus pointing to activity in the DN as a potential cause of performance increases in simple repetitive tasks.

## Introduction

Many tasks in our daily life require that we focus our attention and remain alert over prolonged periods of time. This sustained attention is also referred to as vigilance (Davies and Parasuraman, 1982). For example, vigilance is necessary in busy traffic environments where drivers are required to maintain a high level of attention in order to respond rapidly to critical events in the traffic. Decrements in vigilance are associated with increased error rates (Warm et al., 2008) that could potentially be dangerous in real world scenarios (Molloy and Parasuraman, 1996).

Typical laboratory vigilance tests employ repetitive tasks across long periods, for example the MacWorth’s classic clock test (Mackworth, 1948), where subjects have to monitor the movements of a pointer on a clock and report unpredictable and rare irregularities in how the pointer moves.

Such vigilance tasks are not well suited for fMRI experiments because they provide not enough events for the analysis. The psychomotor vigilance task (PVT) is similar to the classical vigilance tasks. Here subjects also have to respond to the presentation of a simple stimulus, however, these events are more frequent.

The continuous performance task (CPT) is slightly more complex and requires simple perceptual decision making. While performing the task subjects are frequently presented with non-target stimuli (for example a set of consonants) and not so frequently with target stimuli (for example a set of vowels). Subjects are asked to respond to the target stimuli only, and to withhold the response in other cases. In the sustained attention to respond task (SART) the response pattern is inverted. Here subjects are asked to withhold the response to target stimuli but respond to the frequent non-target stimuli.

Both PVT, CPT, and SART have been mostly used to investigate vigilance with fMRI by contrasting blocks of or responses to PVT/CPT/SART performance with blocks of or responses to control tasks that required a lower level of sustained attention (for example repetitively pressing a button).

The described vigilance tasks have been used to reveal that mostly right lateralized frontal cortex, parietal cortex, thalamus and the brain-stem are involved in tasks that require a high level of sustained attention vs. tasks that require a low level of sustained attention (Kinomura et al., 1996; Paus et al., 1997; Sturm et al., 1999; Sturm and Willmes, 2001; Lawrence et al., 2003; Drummond et al., 2005; Raz and Buhle, 2006; Helton et al., 2007, 2010; Lim et al., 2010; Yanaka et al., 2010; Grahn and Manly, 2012; Neale et al., 2015).

However, if the activity of a brain region is associated with tasks that require high levels of sustained attention compared to low level of attention this could simply reflect the different difficulty levels or the workload of the two tasks. In order to confirm that activity in a brain region is really associated with fluctuations of the attentional state the activation has to meet the following two criteria:

(1) The activity has to reflect *trial-by-trial* fluctuations in the performance. The current attentional state will influence the performance during the execution of the task and therefore, activity that reflects the attentional state should also correlate with the performance.

(2) The activity has to *predict* the upcoming performance to a certain degree. Since the attentional state fluctuates slowly, the current level of vigilance will influence the performance in the near future. Activity that reflects the attentional state should be modulated after the execution of the task. Importantly, this modulation should also be observable before the execution of the task.

Here we used a CPT with interspersed prospective memory events in order to identify brain regions that show such a response profile. The prospective memory events were not analyzed here and will be subject of another paper. The vigilance state is reflected in time-varying reaction times (RT). For example it has been shown that sleep deprived (and thus less vigilant) subjects show longer RTs (Doran et al., 2001). It has been shown that lapses of attention are correlated with BOLD responses in prefrontal cortex, thus presumably reflecting reduced vigilance (Weissman et al., 2006). CPTs have recently been used to investigate neural correlates of trial-by-trial fluctuations of vigilance (Esterman et al., 2013, 2014). In a previous study we also could successfully decode RTs in a simple RT experiment. For this we used signals similar to the BOLD signal from the surface of the brain using functional Near Infrared Spectroscopy (Bogler et al., 2014). However, the vigilance decoding was possible after the button presses only. In the present study we increase the spatial resolution using fMRI and cover the whole brain in an unbiased fashion in order to investigate which brain regions are informative about the vigilance state.

## Materials and Methods

### Participants

Twenty-two participants between the ages of 19 and 34 (mean age 25.83, 11 female) took part in the fMRI study. They had normal or corrected-to-normal vision. No participant reported a history of neurological or psychiatric disorders. One participant was excluded due to strong motion (more than 5 mm) during the scanning session. All participants gave informed consent and were compensated with €10 for every hour they participated in the experiment. The study was approved by the ethics committee of the Faculty of Psychology at the Humboldt-Universität zu Berlin (Antrag 2013-34). All subjects gave written informed consent in accordance with the Declaration of Helsinki.

### Design and Procedure

**FIGURE 1:**
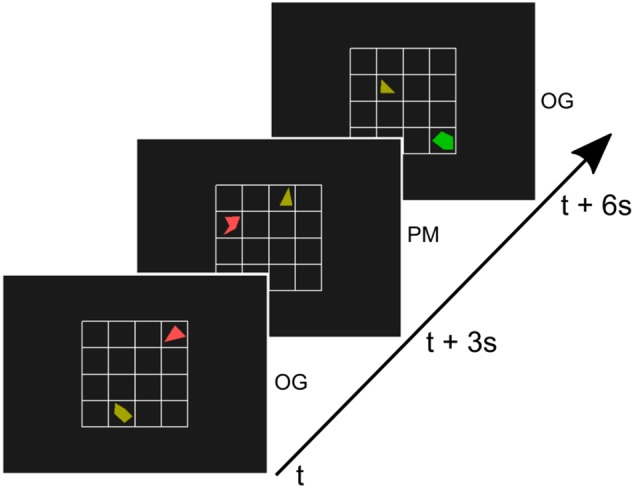
Illustration of three trials of the paradigm. A 4 × 4 grid with a triangle and another random polygon was presented every 3 s for 500 ms. In most trials (90%), the ongoing trials (OG), subjects indicated by button-press whether the non-triangle-shape was positioned left or right to the triangle (adapted from Simons et al., 2006). If the two shapes were a chess knight’s move away from each other (second trial, middle) subjects had to press a different button. These prospective memory (PM) trials were rare (10%).

Subjects were asked to perform a sustained attention task that has previously been used in prospective memory rather than vigilance research and that contains a continued load requiring high attention (Simons et al., 2006). In this task subjects were presented with a small 4 × 4 grid. On this grid two randomly rotated shapes were presented. One of the two shapes was always a triangle, the other one was a random polygon (non-triangle, see **Figure 1**). Each shape was drawn in one of six colors. Irregular shapes were used to avoid recognition at first glance. In most of the trials, participants had to indicate by button-press whether the non-triangle-shape was positioned left or right to the triangle. We will refer to these trials as ongoing trials (OG). However, a different button had to be pressed if the two shapes were a chess knight’s move away from each other (an L shape move, two squares horizontally and one square vertically, or two squares vertically and one square horizontally, see also **Figure 1**). We will refer to these trials as prospective memory trials (PM) (adapted from Simons et al., 2006).

A new configuration of a triangle and polygon was presented every 3 s for a duration of 500 ms with a 2500 ms inter-stimulus-interval. Subjects were asked to respond to the stimuli as soon they were visible and responses were considered valid until the onset of the next stimulus. During each run of the experiment, 204 trials were presented. Twenty trials (9.8%) of the trials involved the PM task. These were randomly distributed across the run with a minimum of 4 OG trials between two PM trials. The first four trials of reach run were always OG trials.

Before the scanning sessions subjects completed three runs of the experiment outside the fMRI scanner in order to familiarize themselves with the task and to avoid training effects during the scanning session. During the fMRI session subjects completed six runs of the experiment. Presentation was controlled and responses were recorded using the Cogent toolbox^1^ for MATLAB 7.0 (The MathWorks, Inc.). Stimuli were projected onto a screen (1024 × 768 pixel, 60 Hz) from the head-end of the scanner.

### fMRI Acquisition

Gradient-echo EPI functional MRI volumes were acquired with a Siemens TRIO 3 T scanner with standard head coil (33 slices, TR = 2000 ms, echo time TE = 30 ms, resolution 3 mm × 3 mm × 3 mm with 0.75 mm gap, FOV 192 mm × 192 mm). In each run 309 images were acquired for each participant. The first three images were discarded to allow for magnetic saturation effects. For every subject six runs of functional MRI were acquired. We also acquired structural MRI data (T1-weighted MPRAGE: 192 sagittal slices, TR = 1900 ms, TE = 2.52 ms, flip angle = 9°, FOV = 256 mm × 256 mm).

### fMRI Preprocessing and Analysis

Data were preprocessed using SPM8.^2^ The functional images were slice time corrected with reference to the first recorded slice, motion corrected, and then spatially smoothed with a Gaussian kernel of 8 mm FWHM. Two analyses were conducted with the preprocessed functional images.

#### Parametric General Linear Model (GLM)

First we used a GLM based fMRI analysis to investigate which individual voxels were activated during the task and furthermore modulated by RT during the trial execution. We assumed a time lag of the BOLD signal by using a canonical haemodynamic response function (HRF). In this analysis we applied a univariate GLM (Friston et al., 1994) to the high pass filtered (cut-off period of 128 s) data of each run. As regressors we used the onsets of the correct and incorrect OG trials and the correct and incorrect PM trials separately that were convolved with a canonical HRF. We also included four parametric regressors with the RT of the correct and incorrect OG and the correct and incorrect PM trials respectively. The parametric regressors were constructed using a parametric modulation of trial onset events with RT. Additionally, six head motion regressors were included as covariates of no interest. Taken together, 14 regressors (4 onset, 4 parametric regressors, and 6 head motion regressors) were used to model the fMRI data. For the group analyses the contrast maps were normalized to a standard stereotaxic space (Montreal Neurological Institute) and re-sampled to an isotropic spatial resolution of 3 mm × 3 mm × 3 mm. Therefore, we co-registered the T1-weighted and the EPI images, applied the unified segmentation algorithm (Ashburner and Friston, 2005) on the T1-weighted image and applied the estimated parameters on the contrast maps. A random effects *t*-test was estimated across subjects in order to test for statistically significant task activation as well as positive and negative parametric modulations of the correct OG trials.

#### Cross-Correlation between RT and fMRI

The second analysis (cross-correlation) extended the first analysis in a way that did not assume any fixed time lag between RT and BOLD signal, therefore we investigated the link between the BOLD-response and RT on longer timescales *before and after* the trial execution. In this analysis we extracted the raw time course for each voxel and each run of a subject and linearly detrended this time course. We then extracted the RTs to the correct OG trials from the same subject. Finally, we calculated the correlation of the BOLD signal with the RT (see **Figure 4** for an illustration of the analysis). Please note, in a control analysis we also used the log(RT) because RTs are not normally distributed (Luce, 1986). These results were almost identical and therefore are not reported. The BOLD signal was sampled with a frequency of 0.5 Hz (TR 2 s: 2, 4, 6, 8, 10, 12 s, ...) and the RT was sampled with a slightly slower frequency of 1/3 Hz (one trial every 3 s: 3, 6, 9, 12 s, ...). In order to be able to calculate the correlation between the two differently sampled time courses we linearly interpolated the RTs to the next matching MR volume. However, we were concerned that we artificially increased the autocorrelation of the RT. So we calculated a control analysis in which we used the subset of the data that was recorded at the same time points. That means for the temporally non-shifted time courses we had 306 volumes and 204 RTs (306 × 2 s = 204 × 3 s = 612 s). Every second RT was recorded at the same time when also a volume was acquired (3, 6, 9, and 12 s, ...). Therefore, the correlation was calculated with a maximum of 102 values (ignoring PM trials). The results of the two analyses were highly similar and here we report the first analyses (with linear interpolation of RTs). The correlation was calculated separately for each of the 6 runs of a subject, Fisher-Z transformed, and averaged across the 6 runs. Importantly, in order to investigate the temporal relationship between brain activity and RTs we repeated this correlation analyses but relatively shifted between -50 s to +50 s in steps of 1 s, ultimately reflecting the cross correlation between the BOLD signal and the RT. For each subject this yielded 101 voxelwise whole-brain maps of Fisher-Z transformed correlation coefficients. These correlation maps were normalized to a standard stereotaxic space (Montreal Neurological Institute) using unified segmentation (see above) and re-sampled to an isotropic spatial resolution of 3 mm × 3 mm × 3 mm. A random effects GLM was estimated for the 101 Fisher-Z normalized correlation coefficients across subjects (ANOVA with one factor time). Final results were estimated using t-contrasts that tested whether the Fisher-Z transformed correlation coefficients of 5 consecutive time lags were significant different from zero.

## Results

### Behavioral Results

**FIGURE 2:**
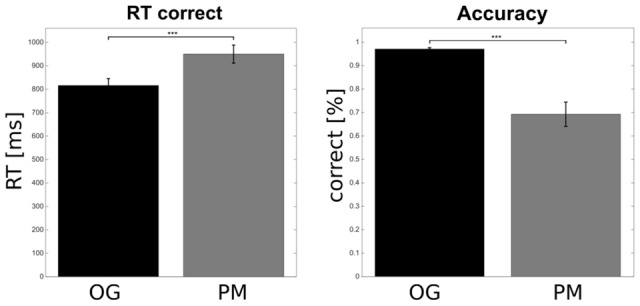
Behavioral results. Participants responded faster to the OG compared to the prospective memory (PM) trials. The accuracy on the OG trials was higher compared to the PM trials. Error bars show the standard error of the mean (^∗∗∗^*p* < 0.001).

**Figure 2** shows the behavioral results. Performance (correct responses) and response times (only for the correct responses) of the OG trials were compared to the PM trials. In total the OG trials were completed with a very high accuracy of 97.09% (SEM = 0.54). The performance on the PM trials with 69.29% (SEM = 5.19) was significantly lower relative to the OG trials [*t*(20) = 5.58, *p* < 0.001]. In addition subjects responded significantly faster in OG trials (mean = 815.89 ms; SEM = 29.64) than in PM trials [mean = 950.08 ms; SEM = 38.44; *t*(20) = -4.8, *p* < 0.001].

We also analyzed the RT of correct OG trials directly preceding PM trials. There was no difference in RT of OG trials preceding correct vs. incorrect PM trials [OG_before_correct_PM_: mean = 805.42 ms; SEM = 30.39; OG_before_incorrect_PM_: mean = 806.14 ms; SEM = 33.02; *t*(20) = -0.06, *p* = 0.95]. The RT of correct PM trials was significant positively correlated with correct OG trials preceding these PM trials [all trials collapsed across subjects: *r* = 0.426; *p* < 0.001; correlation calculated for each subject individually, Fisher-Z normalized and then tested: mean = 0.11; SEM = 0.04; *t*(20) = 2.34, *p* < 0.05].

We conducted an exploratory analysis to investigate whether the performance on the OG trials depends on the similarity to the PM trials. Therefore, we considered the Euclidian distance between the two presented shapes. We calculated the correlation between the absolute difference between the Euclidian distance of the 8 possible OG configurations and the PM configuration and the RT (OG-PM). The correlation was not significantly different from 0 (*r* = -0.4; *p* = 0.32). Taken together the data don’t support the idea that the RT depends on the stimulus configuration of correct OG trials.

### Neuroimaging Analysis 1: Parametric General Linear Model

**FIGURE 3:**
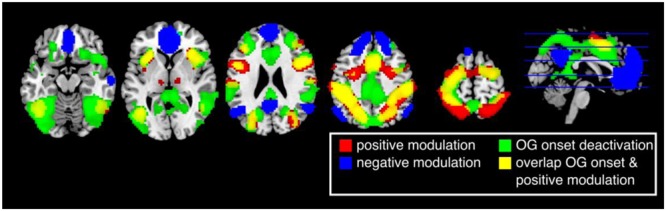
Results of the parametric general linear model. Positively modulated regions (red) are more active when participants respond relatively slow. Negatively modulated regions (blue) are more active when participants respond relatively fast. Brain regions that are deactivated by the task (green) highly overlap with regions that are positive modulated (yellow) (*p* < 0.05, FWE corrected at the voxel level).

The first analysis aimed to reflect previous studies of vigilance and focused on whether canonical BOLD responses elicited by ongoing trials reflected performance fluctuations. Thus, this analysis did not look across longer time scales. Brain responses in the insula, inferior temporal gyrus/V5, middle frontal gyrus, inferior frontal gyrus, supplementary motor area (SMA), postcentral gyrus, inferior parietal lobe, precentral gyrus, early visual cortex, thalamus, and cerebellum were positively modulated by the participants’ response times (**Figure 3**, red and **Table 1**). Thus, these regions were *more active* when participants were *slower* in responding (*p* < 0.05 FWE corrected at the voxel level).

**FIGURE 4:**
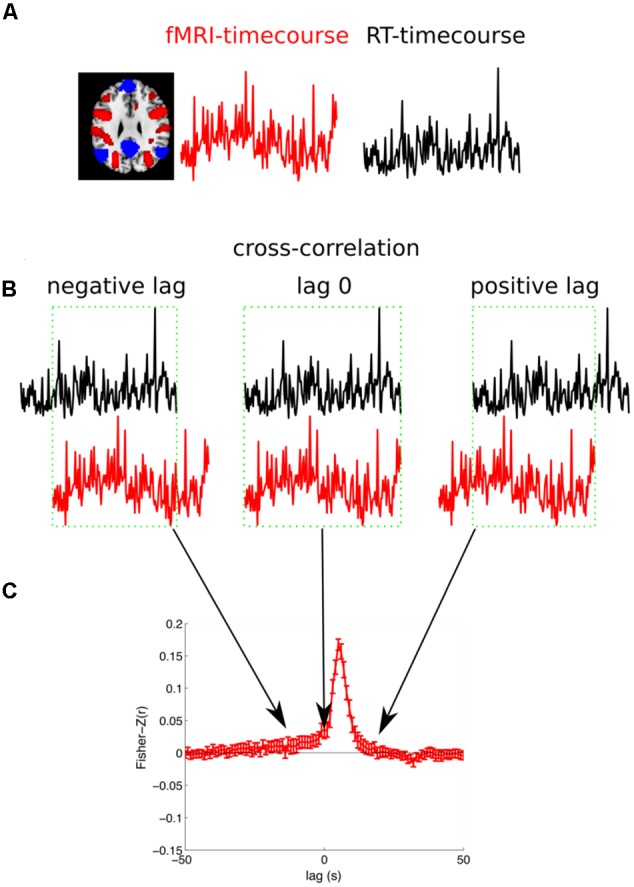
Illustration of the cross-correlation analysis. **(A)** For each subject and each run the fMRI-timecourse for one voxel and the reaction times for the OG trials are extracted. **(B)** The correlation between the fMRI-timecourse and the RT-timecourse is calculated. Therefore, the RT-timecourse is shifted in steps of 1 s in the range from –50 s to +50 s. Here a negative lag (fMRI signal is predictive for behavior), zero lag, and a positive lag (the fMRI signals follows after the behavioral response) are illustrated. The overlapping range, illustrated with a green box, is considered for the calculation of the correlation. **(C)** Finally, the correlation is Fisher-Z transformed. This analysis is repeated for all the six runs for each subject and averaged. The subject specific cross-correlation can then be used for group statistics.

**Table 1:** HRF model; positive modulation *p* < 0.05 (FWE corrected at the voxel level).

Anatomical area	L/R	*T*-value	*Z*-value	*X*	*Y*	*Z*
Insula	R	11.15	Inf	33	20	7
	L	11.98	Inf	–30	17	7
V5; inferior temporal	R	7.52	6.95	48	–58	–8
gyrus	L	9.14	Inf	–39	–61	–5
Middle frontal gyrus	R	6.59	6.19	39	35	16
	L	7.96	7.29	–39	32	28
Inferior frontal gyrus	R	13.29	Inf	48	8	25
	L	12.85	Inf	–48	5	28
Supplementary motor area		10.48	Inf	6	8	49
Postcentral gyrus	R	13.00	Inf	45	–37	49
(Inf parietal lobe)	L	14.90	Inf	–48	–34	46
Inferior parietal lobe	R	12.96	Inf	30	–49	46
	L	14.94	Inf	–33	–43	43
Precentral gyrus; Area 6	R	13.65	Inf	27	–4	52
	L	14.35	Inf	–24	–7	52
Visual cortex; Area 17	R	5.53	5.28	21	–61	4
	L	5.12	4.92	–18	–67	7
Thalamus	R	5.86	5.57	12	–16	–2
	L	5.68	5.41	–12	–19	1
Cerebellum; Lobule VI	R	5.81	5.53	33	–46	–26

Cortical responses in several regions overlapping with the default network, superior medial frontal lobe/ACC, bilateral temporal parietal junction (TPJ), and precuneus, were negatively modulated by response times (*p* < 0.05 FWE corrected at the voxel level). Thus, responses in these regions were *more active* when participants performed *faster*. Other regions with this pattern of modulation included the middle temporal gyrus, middle frontal gyrus, and the cerebellum (**Figure 3**, blue and **Table 2**).

**Table 2:** HRF model; negative modulation *p* < 0.05 (FWE corrected at the voxel level).

Anatomical area	L/R	*T*-value	Z-value	*X*	*Y*	*Z*
Superior medial frontal lobe; ACC		9.52	Inf	–3	56	4
Angular gyrus; TPJ	R	9.06	Inf	54	–64	37
	L	8.39	7.62	–51	–70	34
Precuneus		7.89	7.24	–3	–46	34
Middle temporal gyrus	R	6.40	6.03	63	–16	–14
Middle frontal gyrus	L	4.99	4.80	–39	14	58
Cerebellum; Lobule VIIa Crus I	R	6.60	6.20	33	–82	–35
Cerebellum; Lobule VIIa Crus I	L	6.29	5.93	–30	–79	–32

A number of brain regions showed task dependent deactivation (**Figure 3**, green) that were to a large degree overlapping with regions that showed a positive modulation (**Figure 3**, yellow). None of the areas that were negatively modulated overlapped with regions that showed a task dependent deactivation (*p* < 0.05 FWE corrected at the voxel level).

### Neuroimaging Analysis 2: Cross Correlation between RT and fMRI

**FIGURE 5:**
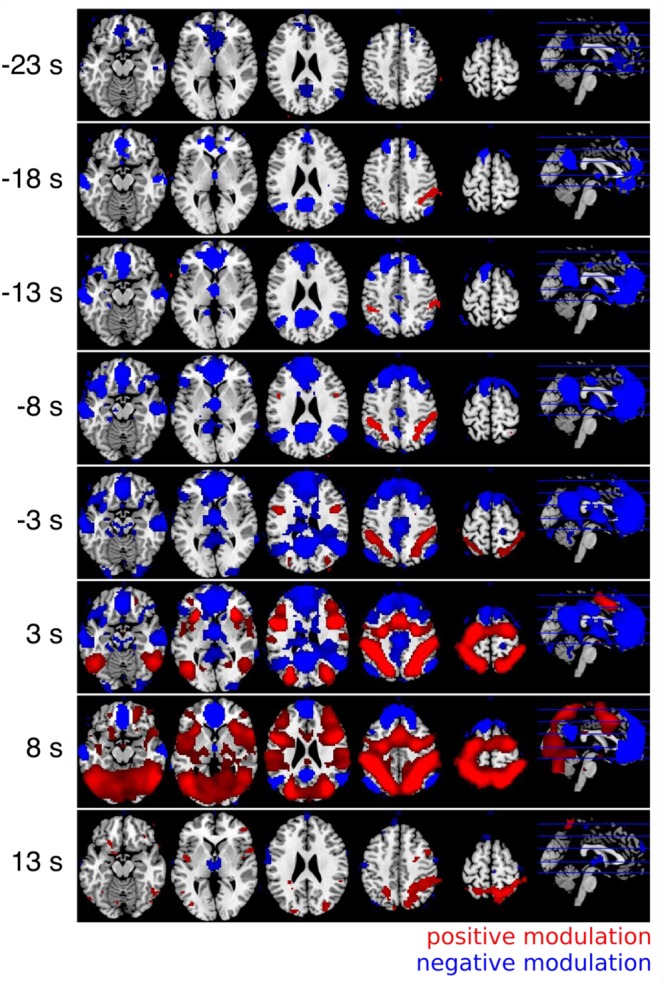
Results of the cross correlation analysis between the (time shifted) fMRI signal and the response times. The Fisher-Z normalized correlation maps with positive lags (3 and 8 s) are very similar to the GLM results (**Figure 3**). The negative correlations can be observed very early up to 18 s *before* the button presses (all *p* < 0.05, FWE corrected at the voxel level). Always five different time lags were combined with t-contrasts and labeled with the mean time lag (i.e., results of the time lags between 1 and 5 s are combined and labeled with a time lag of 3 s in the figure).

The results of the whole brain cross correlation analysis are shown in **Figure 5**. The results were significant with *p* < 0.01 FWE corrected at the voxel level (Bonferroni corrected for 5 tests before stimulus onset). The results of the correlation analysis between the RT and the fMRI response with a positive time lag of a few seconds were very similar to the GLM results. A comparison between the parameter estimates of the GLM for the parametric modulation (Analysis I) and the averaged Fisher-Z transformed correlation coefficients for the 101 different time lags identified a time lag of +5 s as the most similar one (*r* = 0.95). In other words, the voxel time series correlation analysis with a time lag corresponding to the typical lag of the HRF of 5–6 s was almost identical to the standard GLM analysis. Therefore, we can consider how the correlation changes depending on the time lag between the RT and the fMRI response. In **Figure 5** it can be seen that the regions in which the fMRI response is negatively correlated with the RT are informative about the upcoming response times up to 18 s *before* the button presses. The medial prefrontal cortex and the precuneus are informative at first. Regions in which the fMRI response was positively correlated with the RT show the correlation later in time.

**FIGURE 6:**
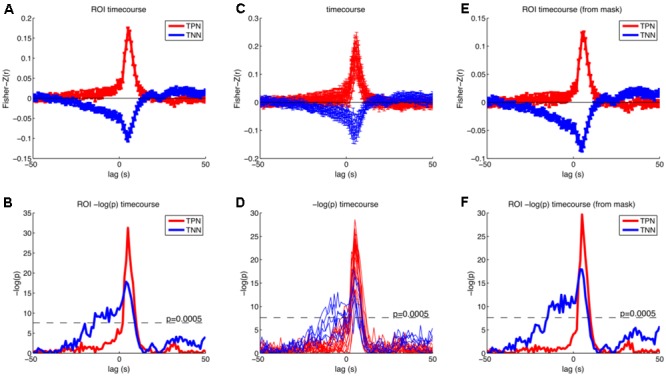
Results of the cross correlation analysis between the (time shifted) fMRI signal and the response times for selected regions. Upper row shows Fisher-Z normalized and averaged correlation coefficients and standard error of the mean for different time lags. Lower row shows –log(p) of the *t*-test on the Fisher-Z normalized correlation coefficients for different time lags. **(A,B)** Timecourses averaged across all reported peak coordinates from the parametric GLM separately for the positive (red) and negative (blue) modulations (**Tables 1, 2**, except cerebellum). **(C,D)** Timecourses separate for all reported peak coordinates from the parametric GLM. Red: positive modulation; Blue: negative modulation. **(E,F)** Timecourses averaged across all voxels that were positively (red) or negatively (blue) modulated with the response time from the parametric GLM with a threshold of *p* < 0.05 (FWE corrected at the voxel level). Correlations rise earlier for the negative correlations and have also higher *p*-values. Task positive network (TPN), task negative network (TNN).

We then further investigated the temporal development of signals in the regions of interest (ROI) obtained by analysis I. We created two masks that contained all positive or all negative modulated voxels (*p* < 0.05; FWE corrected at voxel level). This was done to specifically investigate how the correlation between BOLD and RT developed in regions from which we knew that they were modulated by RT after the button presses. The significant *p*-value was set to 0.0005 in this analysis (Bonferroni corrected for 2 tests and 50 time points before stimulus onset). We also investigated whether the positive and negative modulations developed similar across time. Please note that there is a bias in this analysis for the peak at +5 s (Kriegeskorte et al., 2009). As expected from **Figure 5** the ROI analysis confirmed that the negative correlations arise earlier compared to the positive correlations (see **Figure 6**).

## Discussion

In the present study we implemented a prospective memory task as a type of a CPT to investigate neural correlates of vigilance, as defined by fluctuations of the trial-by-trial performance (RT). We identified two large networks that were modulated by RT, one positively and one negatively. Importantly, RT modulated activity of the default network negatively *before* responses were given. Therefore, despite of some contrary results of previous studies we present evidence that activity in the so-called default network can be associated with better performance in a vigilance task.

The two networks that were modulated by RT had different temporal profiles. **Figure 5** suggests that the negative modulation by RT starts to rise as early as 18–25 s before the trial. However, please note that the autocorrelation of the BOLD-response and the autocorrelation of the behavioral data both lead to an overestimation of this time. Therefore, we don’t want to interpret the absolute time. Instead we want to focus on a relative timing difference between brain regions that show a negative compared to a positive modulation by RT. This comparison is fair because both include the autocorrelation of the BOLD-response and the autocorrelation of the behavioral data. Obviously brain regions that are negatively modulated by RT show this modulation earlier compared to brain regions that are positively modulated by RT.

A large number of brain regions, such as the insula, inferior temporal gyrus, middle frontal gyrus, inferior frontal gyrus, supplementary motor area, visual cortex and parietal cortex, showed a positive modulation with RT. This means, activity in these regions was higher when subjects responded relatively slower. Importantly, the activity in these regions was modulated by RT mostly *after* the button presses, showing a response profile similar like a typical HRF. Possibly, the observed modulation was a simple effect of time-varying signals. That means when more time is spent on signal processing or response preparation, underlying brain regions will show a higher response (Boynton et al., 1996; Dale and Buckner, 1997). It is known, for example, that faster participants show less BOLD activity than slower participants in task-positive brain regions (Haier et al., 1988; Rypma and D’Esposito, 1999; Rypma et al., 2006; Motes et al., 2011). Furthermore, such positive modulations of BOLD activity with RT were reported in similar regions previously (Yarkoni et al., 2009; Grinband et al., 2011; Rao et al., 2014). However, because activity in these regions was mostly modulated *after* the responses, it is unlikely that it reflects the level of task unspecific vigilance.

Brain regions that showed a task dependent deactivation to a large degree overlapped with regions that were also positively modulated by RT. This finding supports the previous interpretation, that regions showing a positive modulation are task specific. In contrast regions that were negatively modulated by RT are candidates for a task independent vigilance modulation.

More interestingly, the default network (DN) (medial frontal lobe/ACC, precuneus, angular gyrus/TPJ) showed a negative modulation with RT. This means, in these regions activity was higher when subjects responded relatively faster. Importantly, activity in these regions was modulated by RT not only after but also *before* the button presses already. In other words, activity in the DN was associated with better performance *before* the execution of the task.

This result might seem surprising because the DN has been defined as a network that showed increased activity in rest conditions compared to different task conditions (Raichle et al., 2001; Raichle and Snyder, 2007). Furthermore, it has been shown that the DN is deactivated during tasks and the degree of deactivation is modulated by task difficulty; the harder the task the stronger the deactivation (McKiernan et al., 2003). Note that in the present study we can not compare activation levels between the task and rest, therefore we can not say whether activity in the DN is higher or lower compared to rest.

In this study we focus on the performance in the OG task. There is the possibility that performance in the OG task is anti-correlated with performance in the PM task, because attention is directed to task demands specific to the OG task or vice versa. RT of OG trials directly preceding PM trials were not different between correct and incorrect PM trials. This result suggests that in our data a pre-error fastening in the PM task (Dudschig and Jentzsch, 2009) was not present. Furthermore, the RT of the OG trials directly preceding PM trials and PM trials are positively correlated. The results suggest that attentional modulation affects both processing of the OG and the PM tasks similarly so that the performance in both tasks improve or decrease synchronously.

Increased activity during episodes of rest or during episodes of decreased performance could be linked to processes such as daydreaming or mind wandering. Mind wandering during the execution of a task could also explain poor performance in the form of increased error rates or increased RTs. Indeed, DN activity has been linked to mind wandering in several studies (Mason et al., 2007; Christoff et al., 2009; Mittner et al., 2014; Fox et al., 2015). Importantly, Mason et al. (2007) also showed that DN activity was higher for practiced compared to novel tasks. The authors argue that during a practiced task the likelihood for mind wandering is higher. However, in the present study it is unlikely that the observed DN activity modulation was linked to mind wandering because activity is higher, when subjects respond faster. During mind wandering slower responses would be expected.

Gilbert et al. (2007) have argued that stimulus-independent thought such as mind wandering is difficult to distinguish from stimulus-oriented thought. In a previous study the same authors have demonstrated higher mPFC (part of the DN) activity for stimulus-oriented compared to stimulus-independent thought (Gilbert et al., 2006). Importantly, in their study mPFC activity was also correlated with RT such that increased mPFC activity was linked to fast responses. Again it is unlikely that this pattern of results is linked to (stimulus-independent) mind wandering because performance should be decreased during episodes of stimulus-independent mind wandering. Another study demonstrated increased DN activity for faster responses under conditions of stimulus unpredictability (Hahn et al., 2007). Taken together, DN activity might support general unfocused monitoring of the external environment rather than internal thought. Indeed, Stawarczyk et al. (2011) found that both internal thoughts and external unfocused attention is associated with activity of midline regions of the DN.

Taken these results together this shows that DN activity seems not exclusively be linked to poor performance. Furthermore, Hampson et al. (2006) demonstrated a positive relationship between the performance during a working memory task and the correlation between two primary nodes of the DN. ACC activity for faster responses could be demonstrated using PET (Naito et al., 2000). Taken together, there is evidence for (rostral) PFC activity during maintaining attention toward the external environment during low demand cognitive tasks (Naito et al., 2000; Small et al., 2003; Gilbert et al., 2006, 2007; Burgess et al., 2007; Hahn et al., 2007).

Furthermore, DN activity has been identified to be linked with good performance in previous sustained attention studies. Esterman et al. (2013, 2014) used another form of a CPT (the gradual onset CPT) and defined good performance as time periods during which subjects showed low variability of RT. Such “in the zone” periods were accompanied with increased activity in the DN. Based on these results Rosenberg et al. (2015) could predict the moment-to-moment attentional state based on activity of the DN, the dorsal attention network and fusiform face area. Note, that Esterman et al. (2013, 2014) did not find areas that were negatively correlated with RT. However, we on the other hand found stronger negative correlations with RT compared to RT variability [abs(RT-mean(RT_run_)]. The different results could be related to the different versions of the CPTs used. In contrast to our experimental design Esterman et al. (2013, 2014) presented visual stimuli with a gradual transition to reduce the potential alerting nature of sudden onsets and offsets.

It has been shown that the strength of the anti-correlation between the DN and the task-positive network mediates behavioral variability in a flanker task (Kelly et al., 2008). In our study, we observed different timecourses of the correlation between network activity and behavioral performance. Therefore, in our task the strength of the anti-correlation between DN and task-positive network seems not related to the behavioral performance, especially not before stimulus onset. The strength of the anti-correlation might be more important during the execution of the task.

Finally, the important role of prefrontal cortex during a vigilance task was directly demonstrated with a transcranial direct current stimulation study (tDCS) (Nelson et al., 2014). TDCS of the prefrontal cortex led to improved behavioral performance as well as improved blood flow velocity and cerebral oxygenation.

We have introduced a new version of a CPT here. Brain regions that modulate vigilance should, in theory, show similar responses for different tasks. Therefore, we think that a variety of different tasks that all require sustained attention are useful in order to identify brain regions that modulate *task independent* vigilance. On the one hand, different tasks will have limitations in the possibility of direct comparisons with each other. On the other hand, only by using a variety of different tasks, the brain network that modulates task independent vigilance can be identified at all.

More research is needed to investigate the circumstances under which DN activity is associated with poor or with good performance. Based on the present findings in combination with previous research we conclude that DN activity is related to good performance if participants are engaged in a demanding sustained attention task during which it is required to monitor the external environment in order to decide which task to perform. Furthermore, during such task requirements DN activity predicts task performance even before the execution of the task. Therefore, it is likely that DN activity reflects the attentional state during certain vigilance tasks.

## Author Contributions

CB planned the study, collected and analyzed the data and wrote the manuscript. AV programmed the experiment, collected data. PZ collected data, analyzed the experiment. J-DH planned the study, wrote the manuscript.

## Conflict of Interest Statement

The authors declare that the research was conducted in the absence of any commercial or financial relationships that could be construed as a potential conflict of interest.

## Footnotes

1 http://www.vislab.ucl.ac.uk/cogent.php

2 http://www.fil.ion.ucl.ac.uk/spm/
